# Environmental and Evolutionary Correlates of Extinction Risk in a Global Biodiversity Hotspot

**DOI:** 10.1002/ece3.74233

**Published:** 2026-08-27

**Authors:** Francis J. Nge, Thi H. Chung

**Affiliations:** ^1^ National Herbarium of New South Wales Botanic Gardens of Sydney Sydney New South Wales Australia; ^2^ SP Jain School of Global Management Lidcombe, Sydney New South Wales Australia

**Keywords:** Australian plants, biodiversity hotspot, diversification rates, extinction risk, machine learning, Rhamnaceae

## Abstract

Macro‐evolutionary and ecological drivers of extinction risk in different organismal groups are not well understood. Some studies have indicated that certain clades may be predisposed to extinction. Through a comparative phylogenetic framework, we assessed whether there are significant evolutionary and environmental correlates of extinction risk for a species rich plant group (Pomaderreae tribe; Rhamnaceae). We focused on the southwest Australian global biodiversity hotspot, where most species of Pomaderreae occur. We also applied 10 different machine learning models to predict and impute data for species that lack flowering time information (18/87 taxa). The *k*‐nearest neighbours algorithm was selected as the best model among the evaluated machine learning approaches because it demonstrated competitive predictive performance, strong recall performance and consistent monthly accuracy for our dataset. We show that larger genera have more threatened species but these species are not over‐represented in larger genera. We also show that extinction risk is uncoupled with evolutionary history (diversification, speciation, extinction rates and lineage age). Shorter flowering periods and temperature variables (WorldClim) were shown to be significant correlates to extinction risk, but polyploidy and precipitation variables were not, even when accounting for phylogenetic relatedness. These findings suggest that threatened species would be more vulnerable to future climate change from their limited ability to respond. The decoupling of diversification rates and lineage age with current extinction risk suggests drivers of extinction risk are multifaceted and are likely predominantly driven by present‐day threats rather than from the past.

## Graphical abstract

We show that larger genera have a greater number of threatened and near‐threatened species, and that extinction risk is uncoupled with evolutionary history (diversification, speciation, extinction rates and lineage age). The decoupling of diversification rates and lineage age with current extinction risk suggests drivers of extinction risk are multifaceted and are likely predominantly driven by present‐day threats rather than from the past.

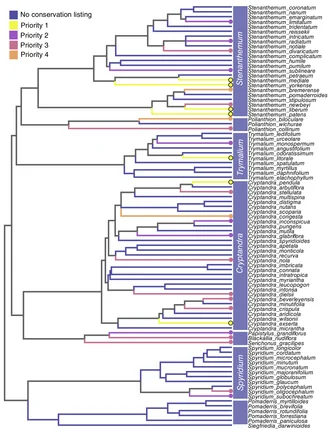

## 1 Introduction

A substantial proportion of vascular plant species (39%–46%) is currently estimated to be threatened with extinction (Nic Lughadha et al. 2020; Bachman et al. 2024; Forest et al. 2026). These threatened species are not evenly distributed across taxonomic groups or geographic regions (Myers et al. 2000; Vamosi and Vamosi 2008; Gray 2019). Lozano and Schwartz (2005) showed that rare plants are over‐represented in large plant families (> 100 taxa), similar to the findings of others (Schwartz and Simberloff 2001). Indeed, families such as Acanthaceae, Gesneriaceae, Piperaceae and Orchidaceae have more than half of their species predicted as being threatened (Bachman et al. 2024), just to name a few. Rare species are shown to be more susceptible to rapid environmental change due to their limited capacity to adapt or migrate (Boyd et al. 2022). However, the evolutionary drivers or predisposition for rarity are understudied.

Studies have shown that certain clades are predisposed to rarity and hence elevated extinction risk (Sjöström and Gross 2006; Fritz and Purvis 2010; Davies et al. 2011; Leao et al. 2014; Malik et al. 2025). However, rarity does not always lead to elevated extinction risk, for example, rare species in remote regions sheltered from disturbance and human impacts. Some of these clades nevertheless show a strong link between selected traits and extinction risk, both correlated with phylogeny (i.e., evolutionary history; Sjöström and Gross 2006; Malik et al. 2025). Traits such as reduced flowering period, fewer seed production, and unisexual breeding systems were correlated with extinction risk (Murray et al. 2002; Sjöström and Gross 2006; Cardillo and Skeels 2016; Dinnage et al. 2020). For example, traits that lead to less reproductive success, dispersal ability and less adaptive potential to threats would amplify extinction risk. Polyploidy is another trait that has been suggested to elevate extinction risk at recent but not deeper evolutionary timescales (Fawcett et al. 2009; Naghiloo and Vamosi 2021). Though autopolyploids have higher extinction rates than allopolyploids (Levin 2019). Many of these traits are linked to the narrow distributional ranges of threatened species (Purvis et al. 2000; Cardillo and Skeels 2016; Chichorro et al. 2019; Sheth et al. 2020). However, these correlates with extinction risk are not always certain, and are shown to be clade‐specific in many instances (Murray et al. 2002; Sheth et al. 2020). In addition, extinction risk is often determined by external threats (e.g., human induced land clearing) and interacts with the responsiveness of intrinsic biotic traits. More studies are required to determine whether there is an emerging consensus on drivers of extinction risk.

Threatened species are unevenly distributed across the globe, with islands as well as biodiversity hotspots and isolated continents such as Australia being over‐represented (Nic Lughadha et al. 2020). The Australian continent contains a diverse flora assemblage, with over 25,000 species, over 80% of which are endemic (Broadhurst and Coates 2017). Concerningly, the continent has one of the highest proportions of assessed plant species classified as threatened across botanical countries globally (Nic Lughadha et al. 2020). 1490 plant taxa are currently listed as threatened nationally under the Environment Protection and Biodiversity Conservation Act 1999 (EPBC Act) as of January 2026. Similarly, in the southwest Australian Floristic Region (SWAFR) global biodiversity hotspot, a high proportion of the flora is rare and threatened (4.3% threatened and 19.5% data deficient; Hopper and Gioia 2004; Gosper et al. 2022). Threatened flora in SWAFR is generally concentrated along coastal and near coastal locations, corresponding with areas of high species richness and endemism within the region (Gosper et al. 2022). These areas include the Swan Coastal Plain, Stirling Range, northern and southern sandplains, Ravensthorpe Range and inland wheatbelt. While studies have focused on human‐induced factors for extinction risk in an Australian context (e.g., Silcock and Fensham 2018), the evolutionary drivers for extinction risk are less well known.

Multiple studies have assessed for areas of phylogenetic diversity (PD) and phylogenetic endemism (PE) across biodiversity hotspots (e.g., Forest et al. 2007; Molina‐Venegas et al. 2017; Qian et al. 2023; Tietje et al. 2023). These studies help guide and prioritise conservation measures across biodiversity hotspots (Forest et al. 2007; Buerki et al. 2015; Daru et al. 2019; Saqib et al. 2025). However, evolutionary drivers for these biodiversity and spatial phylogenetic patterns are less well known. Clades that have higher diversification rates (speciation–extinction) are shown to have higher extinction risk—a pattern seen across different organismal groups (Davies et al. 2011; Greenberg and Mooers 2017; Leão et al. 2020; Tanentzap et al. 2020; Fu et al. 2022). As diversification rates show time dependency in many cases (with fastest rates in the youngest clades; Scholl and Wiens 2016; Henao Diaz et al. 2019), we can argue that younger lineages are also associated with higher extinction risk. Indeed, this phenomenon has been supported in several studies (Davies et al. 2011; Verde Arregoitia et al. 2013; Tanentzap et al. 2020). These associations with higher extinction risk may be linked to higher turnover rates of recently radiated clades (with high diversification rates) and initial smaller geographic range sizes of incipient species (Alfaro et al. 2009; Saupe et al. 2015). The SWAFR biodiversity hotspot contains a large number of narrow endemics and naturally rare plant lineages (Hopper and Gioia 2004; Hopper 2009). The limited distributional ranges of these taxa naturally make them more prone to extinction and thus have higher extinction risk. Based on the findings above, we would expect that narrow endemics with higher diversification rates may have elevated extinction risk than those with lower rates. Untangling potential drivers of extinction risk for threatened taxa is especially timely in order to help conserve the highly species‐rich yet highly threatened biota found in biodiversity hotspots (Myers et al. 2000).

In this study, we selected the Pomaderreae tribe (Rhamnaceae) to explore correlates of rarity and extinction risk in the SWAFR global biodiversity hotspot. The tribe has ~240 species in 10 genera, most of which are found in the southern mesic regions of Australia. All species are woody shrubs or small trees. Rigorous taxonomic work on this tribe over the last few decades has clarified species and generic boundaries, allowing for conservation assessment of taxa without taxonomic confusion (Walsh 1994; Rye 1995a, 1995b, 1996, 2001; Walsh and Coates 1997; Kellermann et al. 2007). Concerningly, a significant portion of Pomaderreae species are already currently listed as of conservation concern, with 16 taxa already listed on the national EPBC threatened flora list and 50 taxa listed on the Priority list of Western Australia (https://www.environment.gov.au, https://florabase.dbca.wa.gov.au/; accessed in July 2026). A robust phylogenomic framework has already been established for the tribe (Nge et al. 2021, 2023, 2024), allowing us to explore evolutionary drivers of extinction risk in a phylogenetic comparative framework. Ploidy level of species included in the phylogeny (215/250 spp.) has also been estimated in a previous study (Nge et al. 2024), allowing us to test whether polyploidy is correlated with extinction risk. For these reasons above, Pomaderreae presents itself as an excellent case study for addressing environmental and evolutionary correlates of extinction risk. Specifically, we address the following questions: (1) whether threatened species are over‐represented in larger genera (Lozano and Schwartz 2005), (2) whether there is significant phylogenetic signal for extinction risk suggesting evolutionary heritability of risk (Fritz and Purvis 2010), (3) whether extinction risk is correlated with higher diversification rates and younger evolutionary age (Davies et al. 2011), and (4) with abiotic (temperature and precipitation environmental variables) or biotic factors (ploidy, flowering period) (Aguilée et al. 2018; Porto and Quental 2026).

## 2 Material and Methods

### 2.1 Dataset Compilation

We quantified extinction risk for each species within the tribe based on the Western Australian (WA) state‐level conservation code (threatened, and Priority 1–4 categories, with Priority 1 being the most threatened among the 4 priority categories) as has been done from other studies (Cardillo and Skeels 2016). These were coded as ordinal variables reflecting the increasing level of conservation concern/priority for each category: 0 = non‐threatened, 4 = Priority four, 3 = Priority three, 2 = Priority two, 1 = Priority one, 5 = Threatened flora. Priority 1 includes species known only from five or less populations on either very small or lands not protected by conservation (i.e., all are under immediate threat). Priority 2 taxa have five or less populations some of which are on lands managed primarily for conservation (i.e., some but not all are under immediate threat). Priority 3 taxa are known from several populations, at least some of which are not under immediate threat. Priority 4 taxa have been adequately surveyed and are naturally rare but not currently threatened. We took a conservative approach and included Priority listed taxa at the species rank and not subspecies or varieties unless all infraspecific rank within a species are priority listed (which none are).

We used the Pomaderreae phylogeny from Nge et al. (2024) for our phylogenetic comparative analyses. The phylogeny included 215 of the 240 species within the tribe. The phylogeny was pruned to contain only WA taxa (*n* = 87) to allow for a robust assessment of phylogenetic signal and evolutionary correlates within WA only. This approach minimises type 1 errors for phylogenetic signal as WA lineages are strongly/entirely clustered in certain clades across the Australasian Pomaderreae phylogeny (Nge et al. 2023, 2024). All 10 genera were still present in the pruned WA phylogeny, and all species except for seven are found in SWAFR. The seven Western Australian taxa included in the phylogeny but not distributed in SWAFR are: *Cryptandra crispula*, *C. intratropica*, *C. monticola* , *C*., *Stenanthemum mediale*, *S. newbeyi*, *S. patens* and *S. petraeum* . Ploidy data, lineage divergence age, diversification tip rates obtained from MiSSE (Vasconcelos et al. 2022), cleaned occurrence records, and associated environmental niche space from WorldClim for Pomaderreae species were sourced from Nge et al. (2024). Lineage age and diversification tip rates were sourced from the full phylogeny instead of the pruned (WA only with 87 taxa) to obtain more accurate metrics, as some WA species/clades are sister to non‐WA clades. We sourced the length of flowering period for each WA species from species profiles in Florabase—the authoritative source (*Flora*) of native plant taxonomic information for Western Australia (https://florabase.dbca.wa.gov.au/; accessed December 2024). Descriptions including flowering time are based on metadata information of vouchered herbarium specimens in WA (Paczkowska and Chapman 2000).

As only 69 of the 87 WA taxa included in our phylogeny had flowering time information, we used machine learning models to predict and impute data for the 18 species that lack flowering time data. For each of the 69 species, flowering time information per month across the 12 months of the year were recorded as binary values (1 for flowering and 0 for not flowering). In addition to flowering time information, we also included the other variables collated above to enhance the prediction models (lineage age, ploidy level, distributional area and length, turnover rates, speciation rates, extinction rates and environmental data from WorldClim). To predict the flowering times for the 18 species, we used the 69‐taxa dataset as the training dataset for 10 different machine learning models: logistic regression, random forest (Liu et al. 2012), support vector machines (Cortes and Vapnik 1995), CatBoost (Prokhorenkova et al. 2018), extra trees (Geurts et al. 2006), LightGBM (Ke et al. 2017), gradient boosting (Friedman 2001), XGBoost (Chen and Guestrin 2016), *k*‐nearest neighbours (KNN; Fix 1985) and multilayer perceptron (MLP) neural networks (Rosenblatt 1957). These models were implemented in Jupyter Notebook using Python v.3.11.13 and machine learning libraries scikit‐learn, XGBoost, LightGBM, CatBoost, TensorFlow and Keras for the MLP model. The models were trained on 69 rows with complete flowering data from the full 87‐taxa dataset (87t) and used to impute missing flowering times for 18 rows, using the following variables: temperature (BIO2, BIO3, BIO4, BIO7), distributional area, length, ploidy, age and WA Priority conservation ranking. Due to the inherently non‐balanced nature of our flowering time dataset, we introduced an additional parameter to account for this in our models (class weight = ‘balanced’). We applied 5‐fold cross‐validation, suitable for the small dataset, to ensure robust accuracy estimates while maximising training data. The models were evaluated using K‐Fold cross‐validation to ensure robust performance. We selected the model with the best of two metrics: (1) the predicting accuracy (high average mean accuracy) and likelihood of errors (low std. dev. mean accuracy). From the best model, hyperparameter tuning was performed.

### 2.2 Data Analyses

To assess whether larger genera do indeed have more species (and over‐representation) with elevated extinction risk, we conducted Spearman rank correlation tests between genus size and number of priority listed species per genus in R v.3.5.1 (R Core Team 2016). To account for phylogenetic relatedness, Phylogenetic Generalised Least Squares (PGLS) tests were conducted using the *caper* v.1.0.4 package in R. The phylogeny was pruned to one tip per genus (*n* = 10) prior to analysis. Results were visualised using the matplotlib.pyplot v.3.10.0 library in Jupyter v.5.8.1 and Plotly's scatter_mapbox in Python v.3.11.13.

To test for phylogenetic signal of extinction risk, we analysed each priority category separately in a binary format (e.g., Priority 1 vs. other) using D‐statistic with 1000 permutations (Fritz and Purvis 2010) from the *caper* v.1.0.2 R package (Orme et al. 2013). Due to the relatively small number of priority listed taxa in each priority category, we also converted the ordinal variables into a binary format (0 = non‐threatened, 1 = threatened) and tested for phylogenetic signal of all threatened vs. non‐threatened taxa. All Priority categories except for Priority 4 (naturally rare but not currently threatened) were converted to ‘1’. We also tested a more conservative threshold and included Priority 4 as threatened due to those taxa been naturally rare and hence having elevated extinction risk than widespread taxa. A D‐stat value of 0 indicates strong phylogenetic signal and value of 1 indicating weak signal (random distribution of traits). Species with a threatened status were also mapped onto the phylogeny via Iroki (Moore et al. 2020).

To test whether extinction risk is correlated with lineage age, diversification rates, abiotic and biotic factors, we conducted phylANOVA for these variables to account for phylogenetic non‐independence, using the R package *phytools* (Revell 2012) in R v.3.5.1 (R Core Team 2016). Adjusted *p*‐values were calculated using the Benjamini–Hochberg method implemented in R. Pairwise post hoc comparisons were also conducted with *p*‐values adjusted for multiple testing using the Holm‐Bonferroni method (*p*.adj = ‘holm’ in *phytools*). We conducted phylANOVA to test for significance between extinction risk and length of flowering time for both the 69 and 87 taxa imputed datasets. We also tested for a phylogenetic signal for length of flowering time (a continuous trait) using Pagel's *λ* (Pagel 1999) and Blomberg's *K* (Blomberg et al. 2003), using *caper* and *ape* (Paradis et al. 2004) packages in R v.3.5.1. The phylogenetic signal was calculated with values between 0 and 1; with 0 indicating no phylogenetic signal and 1 indicating a strong phylogenetic signal (i.e., traits were conserved).

## 3 Results

### 3.1 Extinction Risk Across Western Australia and Clades in Rhamnaceae

**FIGURE 1:**
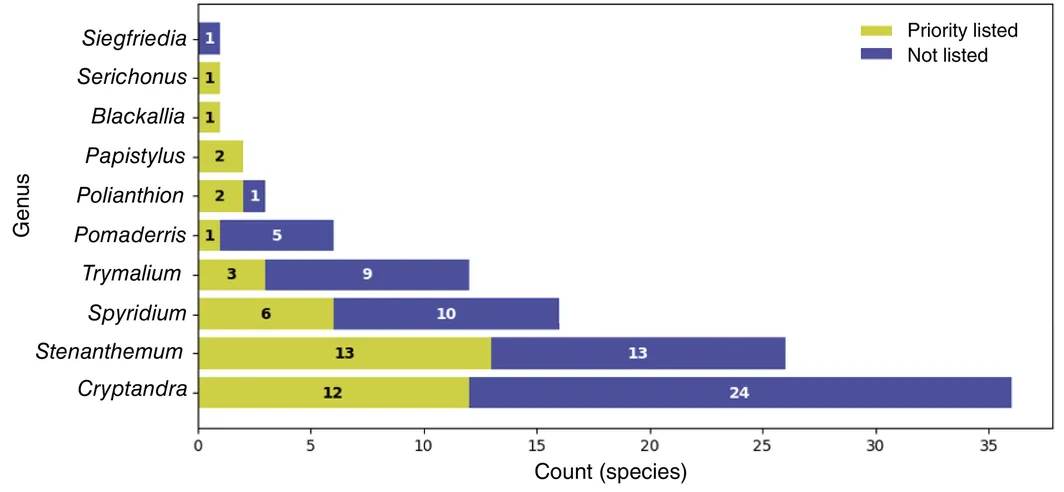
Stacked bar chart showing the proportion of conservation priority‐listed species per genus in Western Australian Pomaderreae (Rhamnaceae).

**FIGURE 2:**
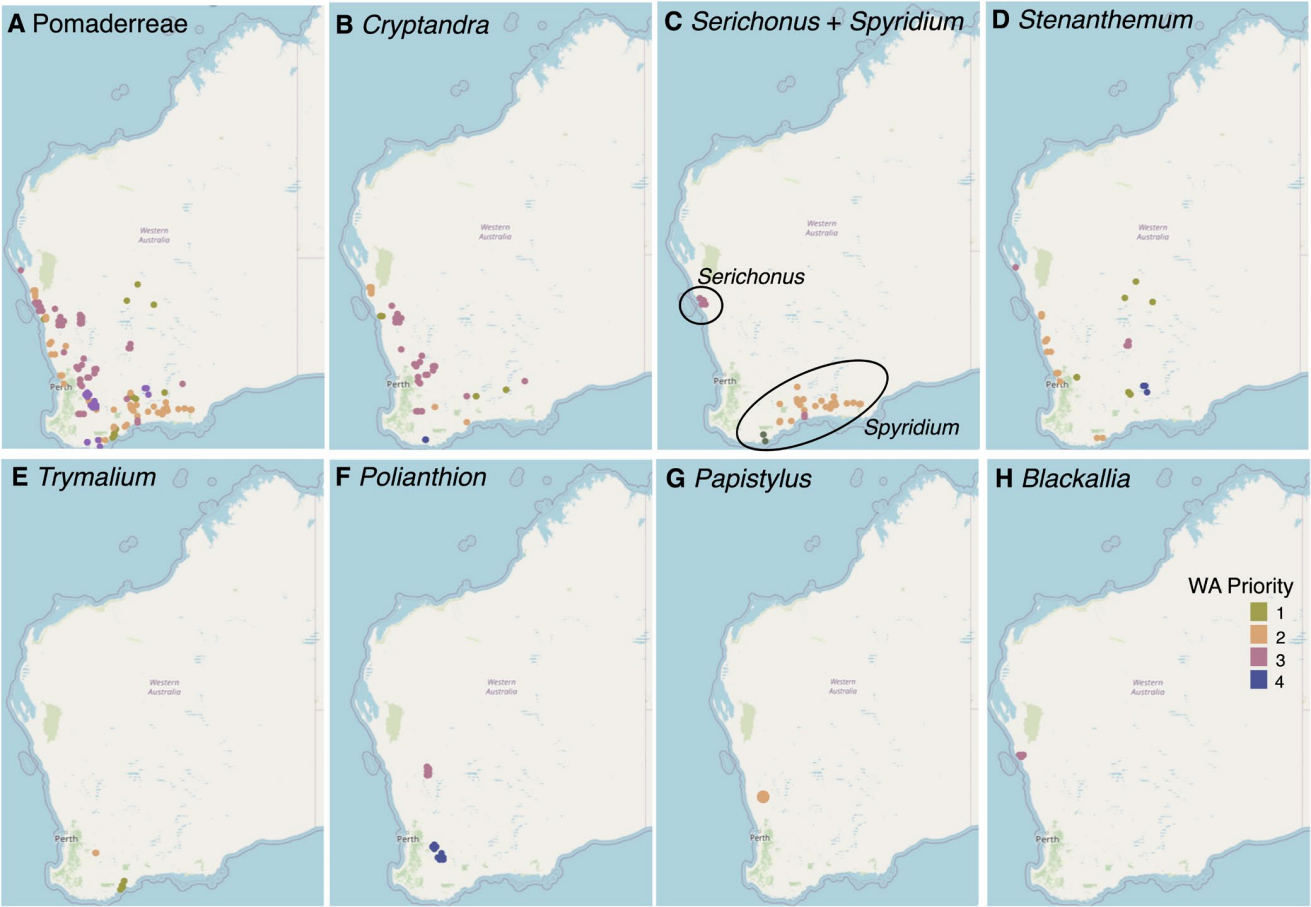
Maps showing the distribution points based on cleaned herbarium records of (A) Western Australian priority listed (yellow) and non‐priority listed (blue) Pomaderreae taxa and priority listed taxa coloured according to the four priority levels: (B) all of Pomaderreae, (B) all of Pomaderreae, (C) *Serichonus* + *Spyridium*, (D) Cryptandra, (E) Stenanthemum, (F) *Polianthion*, (G) *Trymalium*, (H) *Blackallia* + *Papistylus*. The southwest Western Australian Floristic Region (SWAFR) biodiversity hotspot is outlined in red.

We found that the largest genera in Western Australia have the greatest number of threatened species—*Cryptandra* (12/36 spp.), *Stenanthemum* (13/26 spp.), followed by *Spyridium* (6/16 spp.) and *Trymalium* (3/12 spp.) (Spearman *p* < 0.001, rho = 0.89, Figure 1). The majority of the monotypic and small genera also had threatened species in WA (Figure 1). A similar finding was noted from the PGLS test accounting for phylogenetic relatedness (*p* < 0.0001, adjusted *R* ^2^ = 0.89). Priority species of conservation significance are found across the southwest Australian Floristic Region (SWAFR) with no clear ‘hotspot’ centres; however, these species with high extinction risk are notably absent along the Swan Coastal Plain and wet southwest corner of the SWAFR (Figure 2).

### 3.2 Phylogenetic Signal of Rarity

**FIGURE 3:**
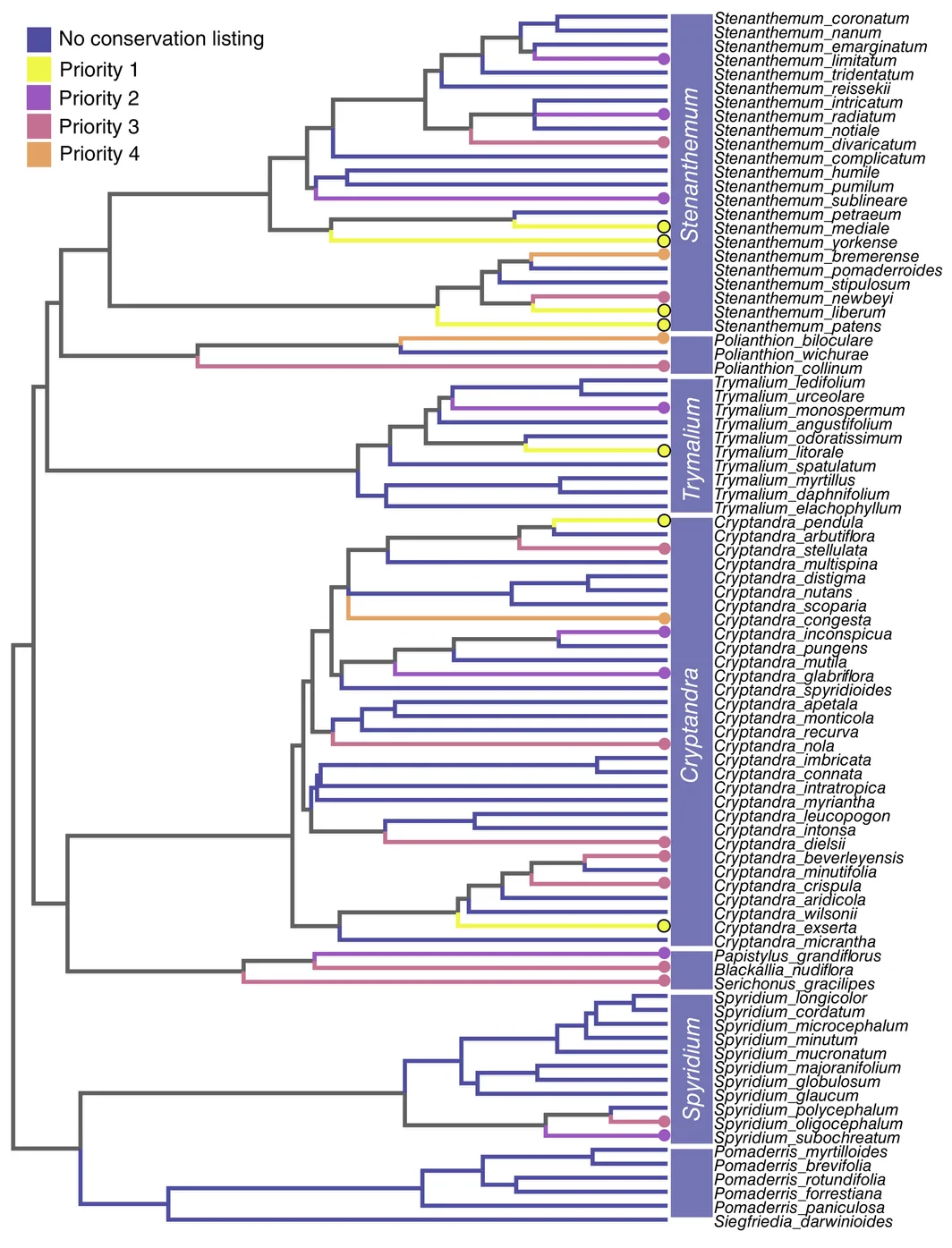
Phylogeny of Western Australian Pomaderreae based on Nge et al. (2024), highlighting Western Australian Priority listed taxa of conservation significance coloured according to the four priority categories.

Our D‐stat results indicate that there was little to no phylogenetic signal for extinction risk across WA Pomaderreae for each Priority category as well as threatened vs. non‐threatened taxa more generally (Table S1). This result was also evident visually in the Pomaderreae phylogeny, with threatened species scattered across clades and all genera except for WA *Pomaderris* (Figure 3).

### 3.3 Machine Learning Model Selection

The K‐Nearest Neighbours (KNN) algorithm was selected among the evaluated machine learning approaches because it demonstrated competitive predictive performance, strong recall performance, and consistent monthly accuracy across the 69 taxa in our dataset. Although Extra Trees achieved the highest overall mean accuracy (69.3%), KNN showed comparable performance (68.9%), differing by only 0.36 percentage points. Given the limited sample size, we prioritised models with strong generalisation ability, reliable detection of flowering events, and consistent performance across flowering periods rather than selecting solely based on marginal differences in overall accuracy.

The macro‐average recall results supported the selection of KNN as the best model. KNN achieved a recall of 0.464, which was higher than Extra Trees (0.425), Random Forest (0.431), CatBoost (0.429), XGBoost (0.418), Gradient Boosting (0.402), LightGBM (0.395) and MLP neural network (0.408). Although Support Vector Machine achieved a slightly higher recall (0.467), KNN provided a stronger balance between recall, overall accuracy, and consistency across flowering months. This indicates that KNN was effective at identifying true flowering events while maintaining stable predictive performance.

To assess whether alternative KNN configurations improved predictive performance, we performed hyperparameter tuning using GridSearchCV, testing neighbour numbers (*k* = 1, 2, 3), weighting schemes and distance metrics (Euclidean and Manhattan). The tuned configuration (*k* = 1, Euclidean distance, uniform weighting) produced lower cross‐validation performance (64.1%) compared with the default KNN configuration (68.9%). The reduced accuracy likely resulted from the small dataset (69 rows) limiting the model's ability to generalise across varied parameter settings and the complex, variable flowering patterns not aligning well with KNN's reliance on local similarity. Therefore, we retained the default KNN model, which uses *k* = 5 neighbours, uniform weighting and Minkowski distance with *p* = 2 (equivalent to Euclidean distance).

The *k* = 5 configuration provides a balance between capturing local similarity among taxa and reducing sensitivity to individual observations. A smaller *k* value, such as *k* = 1, makes predictions highly dependent on individual taxa and may increase susceptibility to noise and overfitting, particularly given the limited training dataset. Conversely, larger neighbourhood sizes may over‐smooth differences among taxa with distinct flowering patterns. Therefore, *k* = 5 was retained as an appropriate compromise between model flexibility and robustness for this dataset. Consequently, from our model selection criteria above, we retained the default KNN model for imputing flowering times for the 18 species.

### 3.4 Correlates of Rarity

**FIGURE 4:**
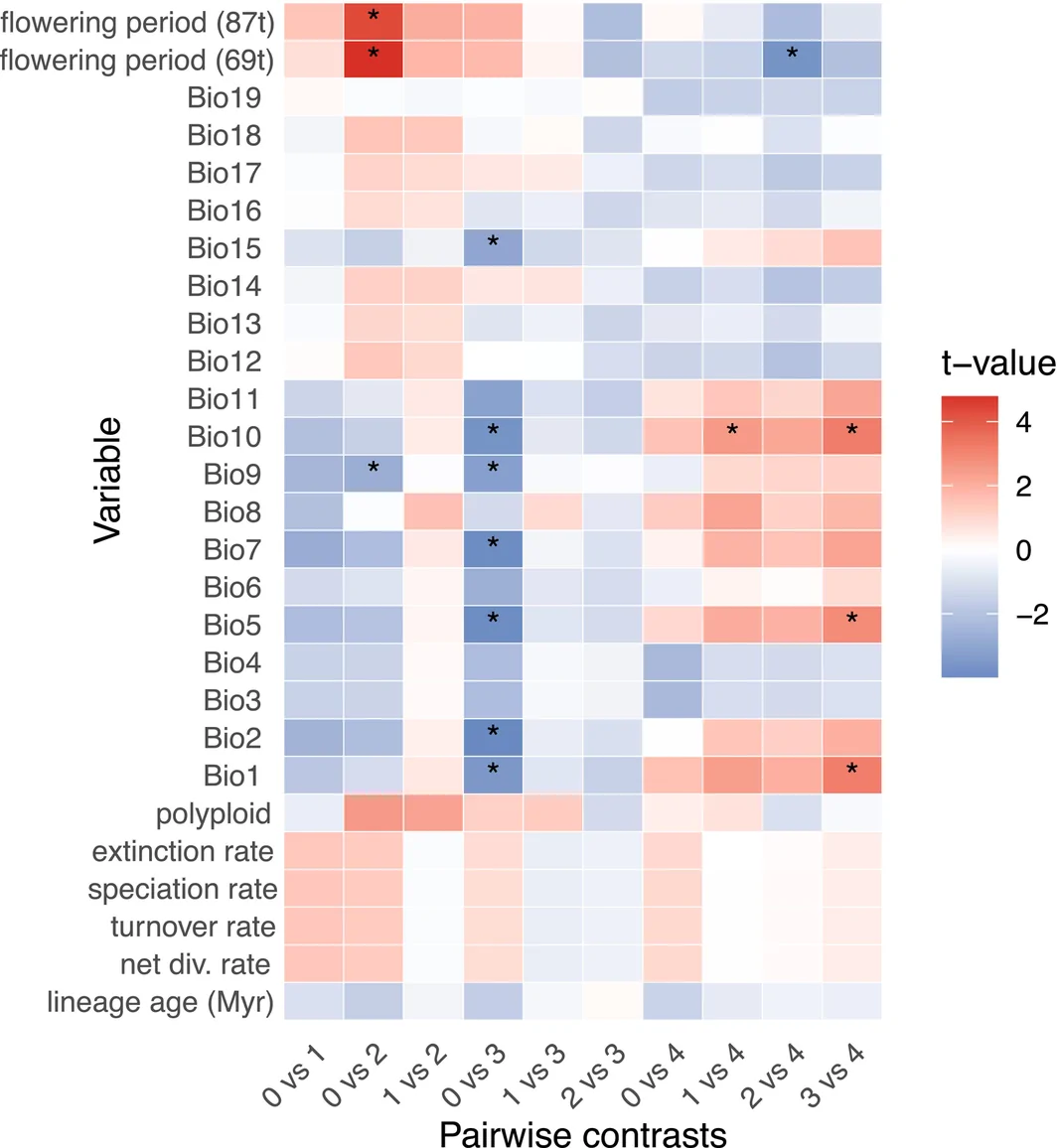
Pairwise post hoc comparison heatmap matrix using the Holm‐Bonferroni method between extinction risk (priority listing) and each included variable. ‘*’ indicates statistical significance (*p* < 0.05). Bio(n) represents WorldClim variables, net div. rate = net diversification rate derived from MiSSE. Flowering period (69t and 87t) is the sum of months across original (69 taxa) and imputed (87t) datasets from machine learning models, respectively. 0 = non‐threatened, and 1 to 4 represents Priority 1 to 4 categories respectively.

Extinction risk was most strongly correlated with temperature variables and not significantly correlated with precipitation variables from our PhylANOVA results (Table 1, Figure 4). We found that extinction risk was not significantly correlated with lineage age, diversification rates (speciation, extinction and turnover rates), and ploidy level (Table 1). We also show that flowering duration (sum of months) was significantly correlated with extinction risk, with threatened species having shorter flowering periods for both original (69t) and imputed (87t) datasets (Table 1). No significant phylogenetic signal was detected for flowering time (sum of months) from both Blomberg *K* and Pagel's *λ* analyses (Table S2).

**TABLE 1:** Summary statistics of correlates with extinction risk accounting for phylogenetic relatedness, based on phylANOVA analyses.

Variable	Sum sq	Residual	*f*	*p* adjusted
Age (Myr)	2.09	25.82	1.64	0.258
Net diversification rate	0.10	2.07	1.02	0.488
Turnover rate	0.10	2.08	1.02	0.488
Speciation rate	0.10	2.07	1.02	0.516
Extinction rate	0.18	3.60	0.99	0.488
Ploidy (binary)	7.42	74.81	2.01	0.196
**BIO1 = Annual Mean Temperature**	0.037	0.16	4.64	0.010**
**BIO2 = Mean Diurnal Range (Mean of monthly (max temp—min temp))**	0.044	0.16	5.55	0.004**
**BIO3 = Isothermality (BIO2/BIO7) (×100)**	0.005	0.03	2.85	0.048*
**BIO4 = Temperature Seasonality (standard deviation ×100)**	0.085	0.35	4.88	0.006**
**BIO5 = Max Temperature of Warmest Month**	0.036	0.13	5.50	0.004**
BIO6 = Min Temperature of Coldest Month	0.083	0.83	2.03	0.178
**BIO7 = Temperature Annual Range (BIO5‐BIO6)**	0.060	0.21	5.76	0.004**
BIO8 = Mean Temperature of Wettest Quarter	0.065	0.70	1.87	0.196
**BIO9 = Mean Temperature of Driest Quarter**	0.056	0.24	4.75	0.009**
**BIO10 = Mean Temperature of Warmest Quarter**	0.037	0.15	5.19	0.004**
**BIO11 = Mean Temperature of Coldest Quarter**	0.038	0.26	2.99	0.049*
BIO12 = Annual Precipitation	0.128	2.43	1.07	0.488
BIO13 = Precipitation of Wettest Month	0.099	3.10	0.65	0.651
BIO14 = Precipitation of Driest Month	0.346	6.17	1.13	0.482
BIO15 = Precipitation Seasonality (Coefficient of Variation)	0.22	1.67	2.65	0.081
BIO16 = Precipitation of Wettest Quarter	0.10	3.11	0.62	0.660
BIO17 = Precipitation of Driest Quarter	0.21	4.73	0.91	0.516
BIO18 = Precipitation of Warmest Quarter	0.26	8.18	0.64	0.651
BIO19 = Precipitation of Coldest Quarter	0.13	3.55	0.72	0.651
**Flowering sum of Months (n) 69taxa**	5.16	12.04	6.86	0.004**
**Flowering sum of Months (n) 87taxa**	4.39	16.74	5.31	0.004**

*Note:* Significant relationships are highlighted in bold and * and ** represent significant *p* values of < 0.05 and < 0.01 respectively. All variables were log‐transformed prior to analyses.

Pairwise post hoc tests revealed differences among conservation categories were non‐linear (Table S3). Significant differences were detected consistently between non‐priority listed (non‐threatened) taxa and Priority 3 listed taxa, with the latter inhabiting more extreme temperature niches—hotter and wider temperature range. A similar pattern was observed between non‐threatened and Priority 2 listed taxa for mean temperature of the warmest quarter (Bio9, Figure 4). Conversely, Priority 4 listed taxa have significantly warmer niches (Bio1, Bio5, Bio10) than Priority 3 and 1 taxa (Figure 4). Precipitation seasonality (Bio15) was detected as significant between non‐threatened and Priority 3 listed taxa; however, this relationship was no longer significant once *p*‐values were adjusted (Table 1, Figure 4). Pairwise comparisons of flowering duration indicated the strongest significant relationship between non‐threatened and Priority 2 listed taxa (Figure 4).

## 4 Discussion

### 4.1 Clade‐Specific and Spatial Patterns of Extinction Risk

The greater number of species with extinction risk (priority listed) present in larger genera of Pomaderreae shown in this study corroborates findings from other plant groups (Schwartz and Simberloff 2001; Lozano and Schwartz 2005). Lozano and Schwartz (2005) showed that mediterranean floras had the greatest proportion of rare species as these floras are dominated by a few exceedingly species rich families (with each containing many rare species). This pattern is also shown in our study for Australian Pomaderreae, as most of the priority listed species are confined to the SWAFR which experiences a mediterranean climate. This tendency for larger genera (or clades) to have a greater number of threatened species may be linked to inherent genetic mechanisms of these clades (e.g., speciation mechanisms and creation of rare distinct lineages) that are worth further investigation.

Our study shows that priority listed species in Western Australia are distributed throughout SWAFR and not concentrated in species‐richness hotspots within SWAFR (Gioia and Hopper 2017). Absence of priority listed Rhamnaceae species across most of the Swan Coastal Plain and southwest corner of SWAFR reflects the paucity of Rhamnaceae species in these regions more generally. The lack of priority listed Rhamnaceae species in the southwestern corner of SWAFR is in contrast to other plant groups such as orchids (Phillips et al. 2011), thus spatial patterns of threatened taxa are likely group‐specific. These contrasting results have important conservation implications and indicate that conservation efforts should not only be focused on areas of high species‐richness but also tailored to specific plant groups as their threatened species are found in different regions (Davies et al. 2011). Future studies on spatial phylogenies could uncover areas of high phylogenetic diversity and endemism and identify whether these hotspots overlap with hotspots of threatened species (Cardillo and Skeels 2016; Mishler 2023; Nge, Biffin, Thornhill, et al. 2025).

### 4.2 Decoupling of Diversification Rates and Extinction Risk

The lack of significant phylogenetic signal for extinction risk in Western Australian Pomaderreae indicates that extinction risk may not be a heritable trait for this plant group. This finding however, cannot be generalised across groups as it is not universal, with a similar finding for *Banksia* (Cardillo and Skeels 2016) but in contrast to others on Australian angiosperms (Sjöström and Gross 2006), *Eucalyptus* (Nytko et al. 2024), the Cape flora (Davies et al. 2011) and tropical plants (Malik et al. 2025). Phylogenetic signal of extinction risk may be limited to broader scale datasets as these studies focused on extinction risk across entire floras or plant families. Similarly, diversification rates and younger lineages were not significantly correlated with extinction risk in Pomaderreae which contradicts the findings of others and suggests that these patterns are likely group‐specific (Davies et al. 2011; Greenberg and Mooers 2017; Tanentzap et al. 2020). This decoupling of rates, lineage age and extinction risk could be attributed to diversification metrics estimated from evolutionary history that are not reflective of present‐day threats. However, we obtained our results even when using diversification tip‐rates (i.e., species‐specific rates; Title and Rabosky 2019) which should give a more accurate estimate of diversification from extant species.

The evolutionary assembly of the extant SWAFR biota over a relatively long and stable period (since the Eocene) may also explain the decoupling of macroevolutionary metrics (rates) and extinction risk. Studies have argued that the SWAFR has been geologically and climatically stable, as the region has not been glaciated since the Permian and experiences oceanic buffering of climate since the Jurassic (Hopper 2009; Harrison and Noss 2017). These conditions were conducive for the gradual accumulation of species in SWAFR over time rather than rapid bursts of recent radiations, which is supported by both paleo‐botanical and phylogenetic research (Cardillo and Pratt 2013; Sniderman et al. 2013; Cook et al. 2015; Nge et al. 2020; Nge and Skeels 2025). Nge et al. (2024) showed that gradual species accumulation and low diversification rates were also the case for Pomaderreae in SWAFR, reinforcing flora‐wide studies. The gradual diversification over long periods of time provides more opportunities for multiple independent convergent evolutionary events to occur, and lower extinction rates in SWAFR would preserve more of these lineages through time. Both these phenomena would likely result in low phylogenetic signal and decoupling of macroevolutionary rates and extinction risk. Indeed, Nge et al. (2023) showed that there were at least eight independent origins of a defence trait (branch spinescence) for *Cryptandra* (Pomaderreae) within SWAFR and surrounding semi‐arid regions. It is plausible that different lineages independently evolved traits that confer higher extinction risk. Additional studies on potential traits linked to extinction risk and across other plant groups are needed to test this hypothesis.

Another explanation for this decoupling is insufficient time for threats to manifest and be detected (extinction debt; Kuussaari et al. 2009). Many of the priority listed Pomaderreae species in WA are found in the agricultural Wheatbelt region, where significant vegetation clearing has occurred within the last hundred years. Less than 10% of the original vegetation remains, mainly as fragmented remnants across the landscape (Wallace and Moore 1987). If threatened Pomaderreae species were driven primarily by habitat loss in the Wheatbelt, then that may also explain the decoupling of extinction risk and evolutionary diversification. Further studies on whether human‐induced threats are directly linked to priority listed/threatened taxa would be required to untangle these alternate hypotheses.

### 4.3 Traits Correlated With Extinction Risk

Overwhelmingly, temperature variables were significant correlates to extinction risk for Pomaderreae in Western Australia. This suggests that not only is temperature an important evolutionary driver of diversification as is shown in this study, the wider Australian flora and other regions (Nge et al. 2020, 2023; Zhou et al. 2024; Nge, Biffin, Rye, et al. 2025; Nge and Skeels 2025), but that it is also important ecologically as a strong correlate for extinction risk, regardless of whether extinction risk is coupled with diversification rates. Our findings that priority listed species occupy more extreme (hotter) temperature niches in SWAFR either suggests they would be pre‐adapted to warmer future climates, or that they would undergo further decline if they are already at the upper physiological limit of temperature tolerance. The stronger statistical significance between non‐threatened and Priority 3 listed taxa might be explained by geography, as all except three Priority 3 taxa are distributed further inland (> 300 km from the coast) with more extreme temperatures. By contrast, non‐threatened taxa are distributed widely, along the coast and across mesic areas of SWAFR. This significant difference between non‐threatened and Priority 3 taxa, as well as no statistical significance between non‐threatened and Priority 4 taxa (naturally rare but not currently threatened) suggests that the threshold between non‐threatened and threatened status is more evident between Priority 3 and 4+ non‐threatened rather than priority listed vs. non‐threatened taxa. Further studies are required to test the validity and generality of this threshold, with conservation implications as it could lead to greater allocation of resources to conserving Priority 1–3 taxa and less to Priority 4 taxa. The lack of statistical significance between the most threatened (Priority 1) and non‐threatened taxa could be due to the inherent nature of our dataset, in which only seven species are recognised as Priority 1 among the sampled 87 WA taxa (compared with 10 classified as Priority 3). Hence, this discrepancy could be due to lack of statistical power resulting in a non‐significant result for Priority 1 taxa even though a more general analysis (all threatened Priority taxa vs. non‐threatened) showed significant differences.

Concerningly, it has been shown repeatedly that projected temperature shifts from climate change would more negatively affect threatened rather than non‐threatened species, due to their restricted geographic range and limited dispersal abilities (Mathes et al. 2021; Duffy et al. 2022). The SWAFR is projected to get warmer and drier as a result of climate change (Hughes 2003). Indeed, forest dieback and decline have been documented within the region in response to increasing drought events (Matusick et al. 2013, 2018). Furthermore, another study indicated that the majority of sampled *Banksia* would undergo range contractions based on these projected climate scenarios (Yates et al. 2010). Thus based on these findings, we surmise that Pomaderreae species with higher extinction risk would also be more severely affected by climate change as many already occupy more extreme environmental niches. Conservation measures to address this threat include species distribution modelling used to inform the translocation of threatened species to more suitable habitats and ex‐situ conservation (Bellis et al. 2021; Casazza et al. 2021).

A shorter flowering period was correlated with extinction risk in Pomaderreae, corroborating studies on Australian Proteaceae (Cardillo and Skeels 2016; Dinnage et al. 2020) and eucalypts (Murray et al. 2002). A shorter flowering period provides less opportunity for successful pollination and hence reproductive success. Shorter flowering period might be linked to smaller geographic range of priority listed species, as wider ranged species would experience different flowering trigger times across its range. Indeed, we show that there is a significant positive correlation between flowering period and area size (spearman rank, rho = 0.38, *p* < 0.001). As flowering period is largely determined by climate (Stephens et al. 2022), a mismatch of flowering activity and pollinator emergence due to climate change is more likely experienced for plants with a shorter flowering period (Forrest 2017; Gérard et al. 2020). Future studies should test whether species with higher extinction risk and a shorter flowering time would be more negatively impacted from climate change. Since flowering is an inherent biological trait, it cannot be manipulated through translocation or ex situ conservation. Rather, field observations on whether there is plant–pollinator mismatch would be required to inform conservation of these threatened species. Further studies should investigate for potential synergistic effects between different traits and threats in the context of future climate change (Davies et al. 2004; Brook et al. 2008).

Other biotic traits such as polyploidy were not significantly correlated with extinction risk in Pomaderreae, likely due to the complex nature of polyploidy, trait adaptation, and conflicting links with extinction risk (Anderson et al. 2023). From a global review of 640 endangered plant species, Pandit et al. (2011) showed that rare and threatened species were disproportionately diploid. However, young polyploidy species are also demonstrated to have unusually high extinction rates due to genomic instability (Levin 2019). But this is not the case for Pomaderreae as shown by Nge et al. (2024) where polyploidy is not significantly correlated with extinction rates or lineage age. Other traits such as apomixis (asexual reproduction through seed) along with other life history traits have been shown to affect extinction risk in plants (Fréville et al. 2007). For *Pomaderris*, a significant proportion of sampled taxa are shown to be apomictic (37.5%, 18/48 taxa) (Chen et al. 2019). Greater sampling is required to assess whether this trait is common across the Pomaderreae tribe (and not just limited to *Pomaderris*), and if it is also significantly correlated with extinction risk.

Many SWAFR plant lineages are also adapted to persisting in naturally fragmented landscapes such as on isolated granite outcrops (Hopper et al. 1997; Byrne and Hopper 2008; Tapper et al. 2014). These studies have documented many rare endemic lineages (with high extinction risk) on specific outcrops, but also very high population divergence among more widespread species, both of which are indicative of low dispersal ability (Hopper 2009). Thus, traits such as dispersal ability rather than diversification rates per se may explain why some lineages have higher extinction risk than others (Crisfield et al. 2024). More broadly, expanded sampling and assessment of other life history traits in addition to the ones included in our study would be important to test for interactive effects and their relationships with extinction risk (Fréville et al. 2007).

The findings presented in this study is based on the current assessment and listing of threatened species which is incomplete by its very nature. We have attempted to account for this by limiting our study focus to Western Australia only, with a relatively well sampled and documented flora (and threats). As each state has its own legislative processes and timeframes for the determination and listing of threatened rare species, a more comprehensive study is required to determine the effects of these factors on evolutionary correlates of extinction risk at continental/national (EPBC), and global levels (International Union for Conservation of Nature: IUCN). Coupling these threat assessments with phylogenetic data under the Evolutionary Distinct and Globally Endangered (EDGE) framework would be a promising next step forward in understanding our threatened flora, with important conservation implications (Gumbs et al. 2023). This EDGE framework can help further prioritise which threatened species require additional attention and monitoring, that is, taxa with a high ED score have high evolutionary and genetic distinctiveness; extinction of these taxa would lead to a disproportionate loss of evolutionary history and diversity.

We also utilised machine learning methods to account for incomplete data, even though the results do not significantly differ between the original and imputed datasets, we showed that this can be a complementary avenue to gap‐fill different data sources (Pollock et al. 2025). Indeed, this is highly relevant for Rhamnaceae as it is one of the families that has a notably high proportion of its species being data‐deficient in terms of threat assessments in SWAFR (Gosper et al. 2022). Machine learning methods have been used to not only gap‐fill trait‐based datasets, but also to predict extinction risk often at a global level (e.g., Bland et al. 2015; Darrah et al. 2017; Nic Lughadha et al. 2020; Borgelt et al. 2022). Encouragingly, these studies have shown the predictions are of high accuracy, for example, up to 91% accuracy in determining extinction risk based on a training dataset of 148 species (Darrah et al. 2017). However, these approaches have limitations, one of which includes the need for large datasets (Ma et al. 2014). Our study used a relatively small training dataset (69 taxa) to impute flowering time information for 18 taxa. Future studies may incorporate flora‐wide datasets or focus on larger plant groups (e.g., eucalypts or *Acacia*, with 700+ species each). New methods for small sample‐size imputation have been developed and are worth exploring further (Bai et al. 2025), expanding on the models that were used in this study.

## 5 Conclusion

In this study, we show that extinction risk of a species‐rich plant group in SWAFR is not evolutionarily inherited and decoupled from macroevolutionary dynamics (diversification, speciation, extinction rates). Instead, present‐day ecological and environmental factors such as more extreme temperatures and shorter flowering time were significant correlates of extinction risk. Several possible hypotheses may explain the decoupling of macroevolution dynamics and present‐day extinction risk, including extinction debt, diversification dynamics within SWAFR and the potential overriding factor of human‐induced threats. Further studies explicitly looking at links between human‐induced threats and extinction risk in a phylogenetic context would be required to address these alternate hypotheses. The lack of strong phylogenetic signal for extinction risk suggests that threats and other causes of rarity are multifaceted and do not just affect one specific clade of plants. The strong correlation of extinction risk with shorter flowering time and temperature variables suggests that priority listed species may be more vulnerable to future climate change due to plant–pollinator mismatch and limited ability to disperse into suitable habitats. Our study and others show that evolutionary and environmental correlates of extinction risk are likely group specific. This has important conservation implications as optimal conservation targets and outcomes would need to be derived from management strategies that are tailored to each specific biotic group. Additional focus should be afforded to group‐specific studies which may provide insights into extinction risk complementing global studies that detect broader general patterns. Increasing adoption of machine learning approaches to address data gaps that are often more pronounced at finer scales would be especially relevant, as more species are increasingly becoming threatened. These approaches, however, also need to be conducted with an informed selection of appropriate datasets of sufficient quality and sample size due to the inherent limitations of these machine learning models.

## Author Contributions

**Francis J. Nge:** conceptualization (lead), data curation (equal), formal analysis (equal), investigation (lead), methodology (equal), project administration (lead), supervision (lead), validation (equal), visualization (lead), writing – original draft (lead), writing – review and editing (lead). **Thi H. Chung:** data curation (equal), formal analysis (equal), investigation (equal), methodology (equal), software (equal), validation (equal), visualization (supporting), writing – original draft (supporting).

## Funding

The authors have nothing to report.

## Conflicts of Interest

The authors declare no conflicts of interest.

## Supporting information


**Table S1:** Summary of the *D*‐statistic tests for phylogenetic signal of extinction risk. The non‐significant *p*‐value for a random expectation indicates that the observed pattern is not strongly different from a random distribution across the phylogeny. The significant *p*‐value (*p* < 0.05) under Brownian structure indicates the trait does not follow a Brownian phylogenetic pattern. NT, non‐threatened.
**Table S2:** Summary statistics of phylogenetic signal for flowering time (sum of months) using Blomberg's *K* and Pagel's lambda.

**Table S3:** Pairwise post hoc tests for each variable with the Priority conservation classification of Western Australia. Label refers to each of the respective variables and corresponding number refer to the sheets in the excel document. The numbers in Group 1 and Group 2 refer to the Priority conservation categories of Western Australia (0 = non‐threatened).

## Data Availability

The dataset and scripts used have been uploaded onto Github and FigShare: https://github.com/Thicabien2004/Plant‐project and https://doi.org/10.6084/m9.figshare.33181115.

## References

[ece374233-bib-0001] Aguilée, R. , F. Gascuel , A. Lambert , and R. Ferriere . 2018. “Clade Diversification Dynamics and the Biotic and Abiotic Controls of Speciation and Extinction Rates.” Nature Communications 9: 1–13. 10.1038/s41467-018-05419-7 PMC6070539 30068945

[ece374233-bib-0002] Alfaro, M. E. , F. Santini , C. Brock , et al. 2009. “Nine Exceptional Radiations Plus High Turnover Explain Species Diversity in Jawed Vertebrates.” Proceedings of the National Academy of Sciences of the United States of America 106: 13410–13414. 19633192 10.1073/pnas.0811087106 PMC2715324

[ece374233-bib-0003] Anderson, B. , J. Pannell , S. Billiard , et al. 2023. “Opposing Effects of Plant Traits on Diversification.” iScience 26: 106362. 37034980 10.1016/j.isci.2023.106362 PMC10074578

[ece374233-bib-0004] Bachman, S. P. , M. J. M. Brown , T. C. C. Leão , E. Nic Lughadha , and B. E. Walker . 2024. “Extinction Risk Predictions for the World's Flowering Plants to Support Their Conservation.” New Phytologist 242: 797–808. 38437880 10.1111/nph.19592

[ece374233-bib-0005] Bai, Y. , Y. Wang , X. Hu , et al. 2025. “Small‐Sample‐Size Trait Imputation Using Deep‐Learning Techniques.” Integrative Zoology: 1–14. 10.1111/1749-4877.70038 41267368

[ece374233-bib-0006] Bellis, J. , M. Longden , J. Styles , and S. Dalrymple . 2021. “Using Macroecological Species Distribution Models to Estimate Changes in the Suitability of Sites for Threatened Species Reintroduction.” Ecological Solutions and Evidence 2: e12050.

[ece374233-bib-0007] Bland, L. M. , B. Collen , C. D. L. Orme , and J. Bielby . 2015. “Predicting the Conservation Status of Data‐Deficient Species.” Conservation Biology 29: 250–259. 25124400 10.1111/cobi.12372

[ece374233-bib-0008] Blomberg, S. P. , T. Garland , and A. R. Ives . 2003. “Testing for Phylogenetic Signal in Comparative Data: Behavioral Traits Are More Labile.” Evolution 57: 717–745. 12778543 10.1111/j.0014-3820.2003.tb00285.x

[ece374233-bib-0009] Borgelt, J. , M. Dorber , M. A. Høiberg , and F. Verones . 2022. “More Than Half of Data Deficient Species Predicted to Be Threatened by Extinction.” Communications Biology 5: 679. 35927327 10.1038/s42003-022-03638-9 PMC9352662

[ece374233-bib-0010] Boyd, J. N. , J. T. Anderson , J. Brzyski , C. Baskauf , and J. Cruse‐Sanders . 2022. “Eco‐Evolutionary Causes and Consequences of Rarity in Plants: A Meta‐Analysis.” New Phytologist 235: 1272–1286. 35460282 10.1111/nph.18172

[ece374233-bib-0011] Broadhurst, L. , and D. Coates . 2017. “Plant Conservation in Australia: Current Directions and Future Challenges.” Plant Diversity 39: 348–356. 30159528 10.1016/j.pld.2017.09.005 PMC6112320

[ece374233-bib-0012] Brook, B. W. , N. S. Sodhi , and C. J. A. Bradshaw . 2008. “Synergies Among Extinction Drivers Under Global Change.” Trends in Ecology & Evolution 23: 453–460. 18582986 10.1016/j.tree.2008.03.011

[ece374233-bib-0013] Buerki, S. , M. W. Callmander , S. Bachman , J. Moat , J.‐N. Labat , and F. Forest . 2015. “Incorporating Evolutionary History Into Conservation Planning in Biodiversity Hotspots.” Philosophical Transactions of the Royal Society, B: Biological Sciences 370: 20140014. 10.1098/rstb.2014.0014 PMC4290428 25561675

[ece374233-bib-0014] Byrne, M. , and S. D. Hopper . 2008. “Granite Outcrops as Ancient Islands in Old Landscapes: Evidence From the Phylogeography and Population Genetics of *Eucalyptus caesia* (Myrtaceae) in Western Australia.” Biological Journal of the Linnean Society 93: 177–188.

[ece374233-bib-0015] Cardillo, M. , and R. Pratt . 2013. “Evolution of a Hotspot Genus: Geographic Variation in Speciation and Extinction Rates in *Banksia* (Proteaceae).” BMC Evolutionary Biology 13: 1471–2148. 10.1186/1471-2148-13-155 PMC3751403 23957450

[ece374233-bib-0016] Cardillo, M. , and A. Skeels . 2016. “Spatial, Phylogenetic, Environmental and Biological Components of Variation in Extinction Risk: A Case Study Using *Banksia* .” PLoS One 11: e0154431. 27148745 10.1371/journal.pone.0154431 PMC4858230

[ece374233-bib-0017] Casazza, G. , T. Abeli , G. Bacchetta , et al. 2021. “Combining Conservation Status and Species Distribution Models for Planning Assisted Colonisation Under Climate Change.” Journal of Ecology 109: 2284–2295.

[ece374233-bib-0018] Chen, S. H. , L. K. Guja , and A. N. Schmidt‐Lebuhn . 2019. “Conservation Implications of Widespread Polyploidy and Apomixis: A Case Study in the Genus *Pomaderris* (Rhamnaceae).” Conservation Genetics 20: 917–926.

[ece374233-bib-0019] Chen, T. , and C. Guestrin . 2016. “Xgboost: A Scalable Tree Boosting System, Proceedings of the 22nd acm sigkdd International Conference on Knowledge Discovery and Data Mining.”

[ece374233-bib-0020] Chichorro, F. , A. Juslén , and P. Cardoso . 2019. “A Review of the Relation Between Species Traits and Extinction Risk.” Biological Conservation 237: 220–229.

[ece374233-bib-0021] Cook, L. G. , N. B. Hardy , and M. D. Crisp . 2015. “Three Explanations for Biodiversity Hotspots: Small Range Size, Geographical Overlap and Time for Species Accumulation. An Australian Case Study.” New Phytologist 207: 390–400. 25442328 10.1111/nph.13199

[ece374233-bib-0022] Cortes, C. , and V. Vapnik . 1995. “Support‐Vector Networks.” Machine Learning 20: 273–297.

[ece374233-bib-0023] Crisfield, V. E. , F. Guillaume Blanchet , C. Raudsepp‐Hearne , and D. Gravel . 2024. “How and Why Species Are Rare: Towards an Understanding of the Ecological Causes of Rarity.” Ecography 2024: e07037.

[ece374233-bib-0024] Darrah, S. E. , L. M. Bland , S. P. Bachman , C. P. Clubbe , and A. Trias‐Blasi . 2017. “Using Coarse‐Scale Species Distribution Data to Predict Extinction Risk in Plants.” Diversity and Distributions 23: 435–447.

[ece374233-bib-0025] Daru, B. H. , P. C. le Roux , J. Gopalraj , D. S. Park , B. G. Holt , and M. Greve . 2019. “Spatial Overlaps Between the Global Protected Areas Network and Terrestrial Hotspots of Evolutionary Diversity.” Global Ecology and Biogeography 28: 757–766.

[ece374233-bib-0026] Davies, K. F. , C. R. Margules , and J. F. Lawrence . 2004. “A Synergistic Effect Puts Rare, Specialized Species at Greater Risk of Extinction.” Ecology 85: 265–271.

[ece374233-bib-0027] Davies, T. J. , G. F. Smith , D. U. Bellstedt , et al. 2011. “Extinction Risk and Diversification Are Linked in a Plant Biodiversity Hotspot.” PLoS Biology 9: e1000620. 21629678 10.1371/journal.pbio.1000620 PMC3101198

[ece374233-bib-0028] Dinnage, R. , A. Skeels , and M. Cardillo . 2020. “Spatiophylogenetic Modelling of Extinction Risk Reveals Evolutionary Distinctiveness and Brief Flowering Period as Threats in a Hotspot Plant Genus.” Proceedings of the Royal Society B: Biological Sciences 287: 20192817. 10.1098/rspb.2019.2817 PMC7282897 32370670

[ece374233-bib-0029] Duffy, K. , T. C. Gouhier , and A. R. Ganguly . 2022. “Climate‐Mediated Shifts in Temperature Fluctuations Promote Extinction Risk.” Nature Climate Change 12: 1037–1044.

[ece374233-bib-0030] Fawcett, J. A. , S. Maere , and Y. Van De Peer . 2009. “Plants With Double Genomes Might Have Had a Better Chance to Survive the Cretaceous–Tertiary Extinction Event.” Proceedings of the National Academy of Sciences of the United States of America 106: 5737–5742. 19325131 10.1073/pnas.0900906106 PMC2667025

[ece374233-bib-0031] Fix, E. 1985. “Discriminatory Analysis: Nonparametric Discrimination, Consistency Properties.” USAF School of Aviation Medicine.

[ece374233-bib-0032] Forest, F. , R. Brown , S. Buerki , et al. 2026. “High Risk of Extinction Across the Flowering Plant Tree of Life.” Science 392: 655–659. 42096575 10.1126/science.adz0773

[ece374233-bib-0033] Forest, F. , R. Grenyer , M. Rouget , et al. 2007. “Preserving the Evolutionary Potential of Floras in Biodiversity Hotspots.” Nature 445: 757–760. 17301791 10.1038/nature05587

[ece374233-bib-0034] Forrest, J. R. K. 2017. “Insect Pollinators and Climate Change.” In Global Climate Change and Terrestrial Invertebrates, edited by S. N. Johnson and T. H. Jones , 69–91. Wiley.

[ece374233-bib-0035] Fréville, H. , K. McConway , M. Dodd , and J. Silvertown . 2007. “Prediction of Extinction in Plants: Interaction of Extrinsic Threats and Life History Traits.” Ecology 88: 2662–2672. 18027768 10.1890/06-1453.1

[ece374233-bib-0036] Friedman, J. H. 2001. “Greedy Function Approximation: A Gradient Boosting Machine.” Annals of Statistics 29: 1189–1232.

[ece374233-bib-0037] Fritz, S. A. , and A. Purvis . 2010. “Selectivity in Mammalian Extinction Risk and Threat Types: A New Measure of Phylogenetic Signal Strength in Binary Traits.” Conservation Biology 24: 1042–1051. 20184650 10.1111/j.1523-1739.2010.01455.x

[ece374233-bib-0038] Fu, Q. , X. Huang , L. Li , et al. 2022. “Linking Evolutionary Dynamics to Species Extinction for Flowering Plants in Global Biodiversity Hotspots.” Diversity and Distributions 28: 2871–2885.

[ece374233-bib-0039] Gérard, M. , M. Vanderplanck , T. Wood , and D. Michez . 2020. “Global Warming and Plant–Pollinator Mismatches.” Emerging Topics in Life Sciences 4: 77–86. 32558904 10.1042/ETLS20190139 PMC7326340

[ece374233-bib-0040] Geurts, P. , D. Ernst , and L. Wehenkel . 2006. “Extremely Randomized Trees.” Machine Learning 63: 3–42.

[ece374233-bib-0041] Gioia, P. , and S. D. Hopper . 2017. “A New Phytogeographic Map for the Southwest Australian Floristic Region After an Exceptional Decade of Collection and Discovery.” Botanical Journal of the Linnean Society 184: 1–15.

[ece374233-bib-0042] Gosper, C. R. , J. M. Percy‐Bower , M. Byrne , T. M. Llorens , and C. J. Yates . 2022. “Distribution, Biogeography and Characteristics of the Threatened and Data‐Deficient Flora in the Southwest Australian Floristic Region.” Diversity 14: 493.

[ece374233-bib-0043] Gray, A. 2019. “The Ecology of Plant Extinction: Rates, Traits and Island Comparisons.” Oryx 53: 424–428.

[ece374233-bib-0044] Greenberg, D. A. , and A. Ø. Mooers . 2017. “Linking Speciation to Extinction: Diversification Raises Contemporary Extinction Risk in Amphibians.” Evolution Letters 1: 40–48. 30283637 10.1002/evl3.4 PMC6121784

[ece374233-bib-0045] Gumbs, R. , C. L. Gray , M. Böhm , et al. 2023. “The EDGE2 Protocol: Advancing the Prioritisation of Evolutionarily Distinct and Globally Endangered Species for Practical Conservation Action.” PLoS Biology 21: e3001991. 36854036 10.1371/journal.pbio.3001991 PMC9974121

[ece374233-bib-0046] Harrison, S. , and R. Noss . 2017. “Endemism Hotspots Are Linked to Stable Climatic Refugia.” Annals of Botany 119: 207–214. 28064195 10.1093/aob/mcw248 PMC5321063

[ece374233-bib-0047] Henao Diaz, L. F. , L. J. Harmon , M. T. Sugawara , E. T. Miller , and M. W. Pennell . 2019. “Macroevolutionary Diversification Rates Show Time Dependency.” Proceedings of the National Academy of Sciences of the United States of America 116: 7403–7408. 30910958 10.1073/pnas.1818058116 PMC6462100

[ece374233-bib-0048] Hopper, S. D. 2009. “OCBIL Theory: Towards an Integrated Understanding of the Evolution, Ecology and Conservation of Biodiversity on Old, Climatically Buffered, Infertile Landscapes.” Plant and Soil 322: 49–86.

[ece374233-bib-0049] Hopper, S. D. , A. P. Brown , and N. G. Marchant . 1997. “Plants of Western Australian Granite Outcrops.” Journal of the Royal Society of Western Australia 80: 141–158.

[ece374233-bib-0050] Hopper, S. D. , and P. Gioia . 2004. “The Southwest Australian Floristic Region: Evolution and Conservation of a Global Hot Spot of Biodiversity.” Annual Review of Ecology, Evolution, and Systematics 35: 623–650.

[ece374233-bib-0051] Hughes, L. 2003. “Climate Change and Australia: Trends, Projections and Impacts.” Austral Ecology 28: 423–443.

[ece374233-bib-0052] Ke, G. , Q. Meng , T. Finley , et al. 2017. “Lightgbm: A Highly Efficient Gradient Boosting Decision Tree.” Advances in Neural Information Processing Systems 30.

[ece374233-bib-0053] Kellermann, J. , B. Rye , and K. Thiele . 2007. “ *Blackallia*, *Serichonus* and *Papistylus*: Three Closely Related Genera of Rhamnaceae (Pomaderreae) From South‐Western Australia.” Nuytsia 16: 299–316.

[ece374233-bib-0054] Kuussaari, M. , R. Bommarco , R. K. Heikkinen , et al. 2009. “Extinction Debt: A Challenge for Biodiversity Conservation.” Trends in Ecology & Evolution 24: 564–571. 19665254 10.1016/j.tree.2009.04.011

[ece374233-bib-0055] Leao, T. C. C. , C. R. Fonseca , C. A. Peres , and M. Tabarelli . 2014. “Predicting Extinction Risk of Brazilian Atlantic Forest Angiosperms.” Conservation Biology 28: 1349–1359. 24665927 10.1111/cobi.12286

[ece374233-bib-0056] Leão, T. C. C. , E. N. Lughadha , and P. B. Reich . 2020. “Evolutionary Patterns in the Geographic Range Size of Atlantic Forest Plants.” Ecography 43: 1510–1520.

[ece374233-bib-0057] Levin, D. A. 2019. “Why Polyploid Exceptionalism Is Not Accompanied by Reduced Extinction Rates.” Plant Systematics and Evolution 305: 1–11.

[ece374233-bib-0058] Liu, Y. , Y. Wang , and J. Zhang . 2012. New Machine Learning Algorithm: Random Forest, International Conference on Information Computing and Applications. Springer.

[ece374233-bib-0059] Lozano, F. D. , and M. W. Schwartz . 2005. “Patterns of Rarity and Taxonomic Group Size in Plants.” Biological Conservation 126: 146–154.

[ece374233-bib-0060] Ma, C. , H. H. Zhang , and X. Wang . 2014. “Machine Learning for Big Data Analytics in Plants.” Trends in Plant Science 19: 798–808. 25223304 10.1016/j.tplants.2014.08.004

[ece374233-bib-0061] Malik, N. , D. Edwards , and R. P. Freckleton . 2025. “Comparative Analyses and Phylogenetic Dependence in Traits and Trends of the Dipterocarpaceae.” Ecology and Evolution 15: e70784. 39803191 10.1002/ece3.70784 PMC11717900

[ece374233-bib-0062] Mathes, G. H. , J. van Dijk , W. Kiessling , and M. J. Steinbauer . 2021. “Extinction Risk Controlled by Interaction of Long‐Term and Short‐Term Climate Change.” Nature Ecology & Evolution 5: 304–310. 33462487 10.1038/s41559-020-01377-w

[ece374233-bib-0063] Matusick, G. , K. X. Ruthrof , N. C. Brouwers , B. Dell , and G. S. J. Hardy . 2013. “Sudden Forest Canopy Collapse Corresponding With Extreme Drought and Heat in a Mediterranean‐Type Eucalypt Forest in Southwestern Australia.” European Journal of Forest Research 132: 497–510.

[ece374233-bib-0064] Matusick, G. , K. X. Ruthrof , J. Kala , N. C. Brouwers , D. D. Breshears , and G. E. S. J. Hardy . 2018. “Chronic Historical Drought Legacy Exacerbates Tree Mortality and Crown Dieback During Acute Heatwave‐Compounded Drought.” Environmental Research Letters 13: 95002.

[ece374233-bib-0065] Mishler, B. D. 2023. “Spatial Phylogenetics.” Journal of Biogeography 50: 1454–1463.

[ece374233-bib-0066] Molina‐Venegas, R. , A. Aparicio , S. Lavergne , and J. Arroyo . 2017. “Climatic and Topographical Correlates of Plant Palaeo‐ and Neoendemism in a Mediterranean Biodiversity Hotspot.” Annals of Botany 119: 229–238. 27288510 10.1093/aob/mcw093 PMC5321055

[ece374233-bib-0067] Moore, R. M. , A. O. Harrison , S. M. McAllister , S. W. Polson , and K. E. Wommack . 2020. “Iroki: Automatic Customization and Visualization of Phylogenetic Trees.” PeerJ 8: e8584. 32149022 10.7717/peerj.8584 PMC7049256

[ece374233-bib-0068] Murray, B. R. , P. H. Thrall , A. M. Gill , and A. B. Nicotra . 2002. “How Plant Life‐History and Ecological Traits Relate to Species Rarity and Commonness at Varying Spatial Scales.” Austral Ecology 27: 291–310.

[ece374233-bib-0069] Myers, N. , R. A. Mittermeier , C. G. Mittermeier , G. A. Da Fonseca , and J. Kent . 2000. “Biodiversity Hotspots for Conservation Priorities.” Nature 403: 853–858. 10706275 10.1038/35002501

[ece374233-bib-0070] Naghiloo, S. , and J. C. Vamosi . 2021. “Correlates of Extinction Vulnerability in Canadian's Prairie Ecoregion.” Biodiversity and Conservation 30: 2495–2509.

[ece374233-bib-0071] Nge, F. J. , E. Biffin , B. L. Rye , et al. 2025. “Australian Biogeography, Climate‐Dependent Diversification and Phylogenomics of the Spectacular Chamelaucieae Tribe (Myrtaceae).” Australian Systematic Botany 38: SB24014.

[ece374233-bib-0072] Nge, F. J. , E. Biffin , K. R. Thiele , and M. Waycott . 2020. “Extinction Pulse at Eocene–Oligocene Boundary Drives Diversification Dynamics of the Two Australian Temperate Floras.” Proceedings of the Royal Society B: Biological Sciences 287: 20192546. 10.1098/rspb.2019.2546 PMC7015341 31964242

[ece374233-bib-0073] Nge, F. J. , E. Biffin , A. H. Thornhill , K. van Dijk , and M. Waycott . 2025. “Phylogenomic Incongruence Leads to Different Spatial Phylogenetic Patterns: An Australian Continental Scale Case Study With *Isopogon* and *Petrophile* (Proteaceae).” Australian Systematic Botany 38: SB25001.

[ece374233-bib-0074] Nge, F. J. , T. A. Hammer , T. Vasconcelos , E. Biffin , J. Kellermann , and M. Waycott . 2024. “Polyploidy Linked With Species Richness but Not Diversification Rates or Niche Breadth in Australian Pomaderreae (Rhamnaceae).” Annals of Botany 3: 531–548. 10.1093/aob/mcae181 PMC11920800 39441970

[ece374233-bib-0075] Nge, F. J. , J. Kellermann , E. Biffin , K. R. Thiele , and M. Waycott . 2023. “Rise and Fall of a Continental Mesic Radiation in Australia: Spine Evolution, Biogeography, and Diversification of *Cryptandra* (Rhamnaceae: Pomaderreae).” Botanical Journal of the Linnean Society 204: 327–342.

[ece374233-bib-0076] Nge, F. J. , J. Kellermann , E. Biffin , M. Waycott , and K. R. Thiele . 2021. “Historical Biogeography of *Pomaderris* (Rhamnaceae): Continental Vicariance in Australia and Repeated Independent Dispersals to New Zealand.” Molecular Phylogenetics and Evolution 158: 107085. 33540078 10.1016/j.ympev.2021.107085

[ece374233-bib-0077] Nge, F. J. , and A. Skeels . 2025. “Diversification Patterns of the Southwest Australian Biodiversity Hotspot Reveal a Novel Macroevolutionary Pathway to Plant Hyperdiversity.” New Phytologist 248: 953–967. 40589033 10.1111/nph.70330 PMC12445814

[ece374233-bib-0078] Nic Lughadha, E. , S. P. Bachman , T. C. C. Leão , et al. 2020. “Extinction Risk and Threats to Plants and Fungi.” Plants, People, Planet 2: 389–408.

[ece374233-bib-0079] Nytko, A. G. , J. K. Senior , R. C. Wooliver , J. O'Reilly‐Wapstra , J. A. Schweitzer , and J. K. Bailey . 2024. “An Evolutionary Case for Plant Rarity: *Eucalyptus* as a Model System.” Ecology and Evolution 14: e11440. 38855318 10.1002/ece3.11440 PMC11156952

[ece374233-bib-0080] Orme, D. , R. Freckleton , G. Thomas , et al. 2013. “The Caper Package: Comparative Analysis of Phylogenetics and Evolution in R.” R Package Version 3.5.1.

[ece374233-bib-0081] Paczkowska, G. , and A. R. Chapman . 2000. The Western Australian Flora: A Descriptive Catalogue. Wildflower Society of Western Australia: Western Australian Herbarium, CALM.

[ece374233-bib-0082] Pagel, M. 1999. “Inferring the Historical Patterns of Biological Evolution.” Nature 401: 877–884. 10553904 10.1038/44766

[ece374233-bib-0083] Pandit, M. K. , M. J. O. Pocock , and W. E. Kunin . 2011. “Ploidy Influences Rarity and Invasiveness in Plants.” Journal of Ecology 99: 1108–1115.

[ece374233-bib-0084] Paradis, E. , J. Claude , and K. Strimmer . 2004. “APE: Analyses of Phylogenetics and Evolution in R Language.” Bioinformatics 20: 289–290. 14734327 10.1093/bioinformatics/btg412

[ece374233-bib-0085] Phillips, R. D. , A. P. Brown , K. W. Dixon , and S. D. Hopper . 2011. “Orchid Biogeography and Factors Associated With Rarity in a Biodiversity Hotspot, the Southwest Australian Floristic Region.” Journal of Biogeography 38: 487–501.

[ece374233-bib-0086] Pollock, L. J. , J. Kitzes , S. Beery , et al. 2025. “Harnessing Artificial Intelligence to Fill Global Shortfalls in Biodiversity Knowledge.” Nature Reviews Biodiversity 1: 1–17.

[ece374233-bib-0087] Porto, L. M. V. , and T. B. Quental . 2026. “High Competition and Selective Extinction: How Biotic and Abiotic Drivers Shaped Speciation and Extinction Regimes in Carnivora.” Evolution 80: 936–949. 41604196 10.1093/evolut/qpag011

[ece374233-bib-0088] Prokhorenkova, L. , G. Gusev , A. Vorobev , A. V. Dorogush , and A. Gulin . 2018. “CatBoost: Unbiased Boosting With Categorical Features.” Advances in Neural Information Processing Systems 31.

[ece374233-bib-0089] Purvis, A. , J. L. Gittleman , G. Cowlishaw , and G. M. Mace . 2000. “Predicting Extinction Risk in Declining Species.” Proceedings of the Royal Society of London. Series B: Biological Sciences 267: 1947–1952. 10.1098/rspb.2000.1234 PMC1690772 11075706

[ece374233-bib-0090] Qian, H. , J. Zhang , and M. Jiang . 2023. “Global Patterns of Taxonomic and Phylogenetic Diversity of Flowering Plants: Biodiversity Hotspots and Coldspots.” Plant Diversity 45: 265–271. 37397596 10.1016/j.pld.2023.01.009 PMC10311147

[ece374233-bib-0091] R Core Team . 2016. R: A Language and Environment for Statistical Computing. R Foundation for Statistical Computing.

[ece374233-bib-0092] Revell, L. J. 2012. “Phytools: An R Package for Phylogenetic Comparative Biology (and Other Things).” Methods in Ecology and Evolution 3: 217–223.

[ece374233-bib-0093] Rosenblatt, F. 1957. The Perceptron, A Perceiving and Recognizing Automaton Project Para. Cornell Aeronautical Laboratory.

[ece374233-bib-0094] Rye, B. 1995a. “New and Priority Taxa in the Genera *Cryptandra* and *Stenanthemum* (Rhamnaceae) of Western Australia.” Nuytsia 10: 255–305.

[ece374233-bib-0095] Rye, B. 1995b. “New and Priority Taxa in the Genera *Spyridium* and *Trymalium* (Rhamnaceae) of Western Australia.” Nuytsia 10: 119–140.

[ece374233-bib-0096] Rye, B. 1996. “ *Granitites*, a New Genus of Rhamnaceae From the South‐West of Western Australia.” Nuytsia—The Journal of the Western Australian Herbarium 10: 451–457.

[ece374233-bib-0097] Rye, B. 2001. “A Taxonomic Update of *Stenanthemum* (Rhamnaceae: Pomaderreae) in Western Australia.” Nuytsia—The Journal of the Western Australian Herbarium 13: 495–507.

[ece374233-bib-0098] Saqib, S. , F. Ullah , W. O. Omollo , et al. 2025. “Identifying Hotspots and Climate Drivers of Alien Plant Species for Conservation Prioritization Across the Pan‐Himalaya.” Biological Conservation 302: 110994.

[ece374233-bib-0099] Saupe, E. E. , H. Qiao , J. R. Hendricks , et al. 2015. “Niche Breadth and Geographic Range Size as Determinants of Species Survival on Geological Time Scales.” Global Ecology and Biogeography 24: 1159–1169.

[ece374233-bib-0100] Scholl, J. P. , and J. J. Wiens . 2016. “Diversification Rates and Species Richness Across the Tree of Life.” Proceedings of the Royal Society B: Biological Sciences 283: 20161334. 10.1098/rspb.2016.1334 PMC5031659 27605507

[ece374233-bib-0101] Schwartz, M. W. , and D. Simberloff . 2001. “Taxon Size Predicts Rates of Rarity in Vascular Plants.” Ecology Letters 4: 464–469.

[ece374233-bib-0102] Sheth, S. N. , N. Morueta‐Holme , and A. L. Angert . 2020. “Determinants of Geographic Range Size in Plants.” New Phytologist 226: 650–665. 31901139 10.1111/nph.16406

[ece374233-bib-0103] Silcock, J. L. , and R. J. Fensham . 2018. “Using Evidence of Decline and Extinction Risk to Identify Priority Regions, Habitats and Threats for Plant Conservation in Australia.” Australian Journal of Botany 66: 541–555.

[ece374233-bib-0104] Sjöström, A. , and C. L. Gross . 2006. “Life‐History Characters and Phylogeny Are Correlated With Extinction Risk in the Australian Angiosperms.” Journal of Biogeography 33: 271–290.

[ece374233-bib-0105] Sniderman, J. M. K. , G. J. Jordan , and R. M. Cowling . 2013. “Fossil Evidence for a Hyperdiverse Sclerophyll Flora Under a Non–Mediterranean‐Type Climate.” Proceedings of the National Academy of Sciences of the United States of America 110: 3423–3428. 23401515 10.1073/pnas.1216747110 PMC3587219

[ece374233-bib-0106] Stephens, R. E. , H. Sauquet , G. R. Guerin , M. Jiang , D. Falster , and R. V. Gallagher . 2022. “Climate Shapes Community Flowering Periods Across Biomes.” Journal of Biogeography 49: 1205–1218.

[ece374233-bib-0107] Tanentzap, A. J. , J. Igea , M. G. Johnston , and M. J. Larcombe . 2020. “Does Evolutionary History Correlate With Contemporary Extinction Risk by Influencing Range Size Dynamics?” American Naturalist 195: 569–576. 10.1086/707207 32097046

[ece374233-bib-0108] Tapper, S. L. , M. Byrne , C. J. Yates , et al. 2014. “Prolonged Isolation and Persistence of a Common Endemic on Granite Outcrops in Both Mesic and Semi‐Arid Environments in South‐Western Australia.” Journal of Biogeography 41: 2032–2044.

[ece374233-bib-0109] Tietje, M. , A. Antonelli , F. Forest , et al. 2023. “Global Hotspots of Plant Phylogenetic Diversity.” New Phytologist 240: 1636–1646. 37496281 10.1111/nph.19151

[ece374233-bib-0110] Title, P. O. , and D. L. Rabosky . 2019. “Tip Rates, Phylogenies and Diversification: What Are We Estimating, and How Good Are the Estimates?” Methods in Ecology and Evolution 10: 821–834.

[ece374233-bib-0111] Vamosi, J. C. , and S. M. Vamosi . 2008. “Extinction Risk Escalates in the Tropics.” PLoS One 3: e3886. 19066623 10.1371/journal.pone.0003886 PMC2586650

[ece374233-bib-0112] Vasconcelos, T. , B. C. O'Meara , and J. M. Beaulieu . 2022. “A Flexible Method for Estimating Tip Diversification Rates Across a Range of Speciation and Extinction Scenarios.” Evolution 76: 1420–1433. 35661352 10.1111/evo.14517

[ece374233-bib-0113] Verde Arregoitia, L. D. , S. P. Blomberg , and D. O. Fisher . 2013. “Phylogenetic Correlates of Extinction Risk in Mammals: Species in Older Lineages Are Not at Greater Risk.” Proceedings of the Royal Society B: Biological Sciences 280: 20131092. 10.1098/rspb.2013.1092 PMC3712450 23825210

[ece374233-bib-0114] Wallace, K. J. , and S. A. Moore . 1987. “Management of Remnant Bushland for Nature Conservation in Agricultural Areas of Southwestern Australia—Operational and Planning Perspectives.” In Nature Conservation: The Role of Remnants of Native Vegetation, edited by D. A. Saunders , G. W. Arnold , A. Burbidge , and A. J. M. Hopkins , 259–268. Surrey Beatty.

[ece374233-bib-0115] Walsh, N. 1994. “Pomaderris Brevifolia (Rhamnaceae): A New Species From South‐West Western Australia.” Muelleria: An Australian Journal of Botany 8: 107–111.

[ece374233-bib-0116] Walsh, N. , and F. Coates . 1997. “New Taxa, New Combinations and an Infrageneric Classification in *Pomaderris* (Rhamnaceae).” Muelleria: An Australian Journal of Botany 10: 27–56.

[ece374233-bib-0117] Yates, C. J. , A. McNeill , J. Elith , and G. F. Midgley . 2010. “Assessing the Impacts of Climate Change and Land Transformation on Banksia in the South West Australian Floristic Region.” Diversity and Distributions 16: 187–201.

[ece374233-bib-0118] Zhou, L. , K. Ma , L. Zhu , et al. 2024. “Spatial Patterns and Predictors of Seed Plants' Extinction Risks in Asian Countries.” Biological Conservation 289: 110424.

