# Correction to “Environmental and Evolutionary Correlates of Extinction Risk in a Global Biodiversity Hotspot”

**DOI:** 10.1002/ece3.74349

**Published:** 2026-09-10

Nge, F. J. , and T. H. Chung . 2026. “Environmental and Evolutionary Correlates of Extinction Risk in a Global Biodiversity Hotspot.” Ecology and Evolution 16, no. 9: e74233. 10.1002/ece3.74233.42662516 PMC13519961

Figure 2 was incorrect. The correct Figure 2 is provided below.

**FIGURE 2:**
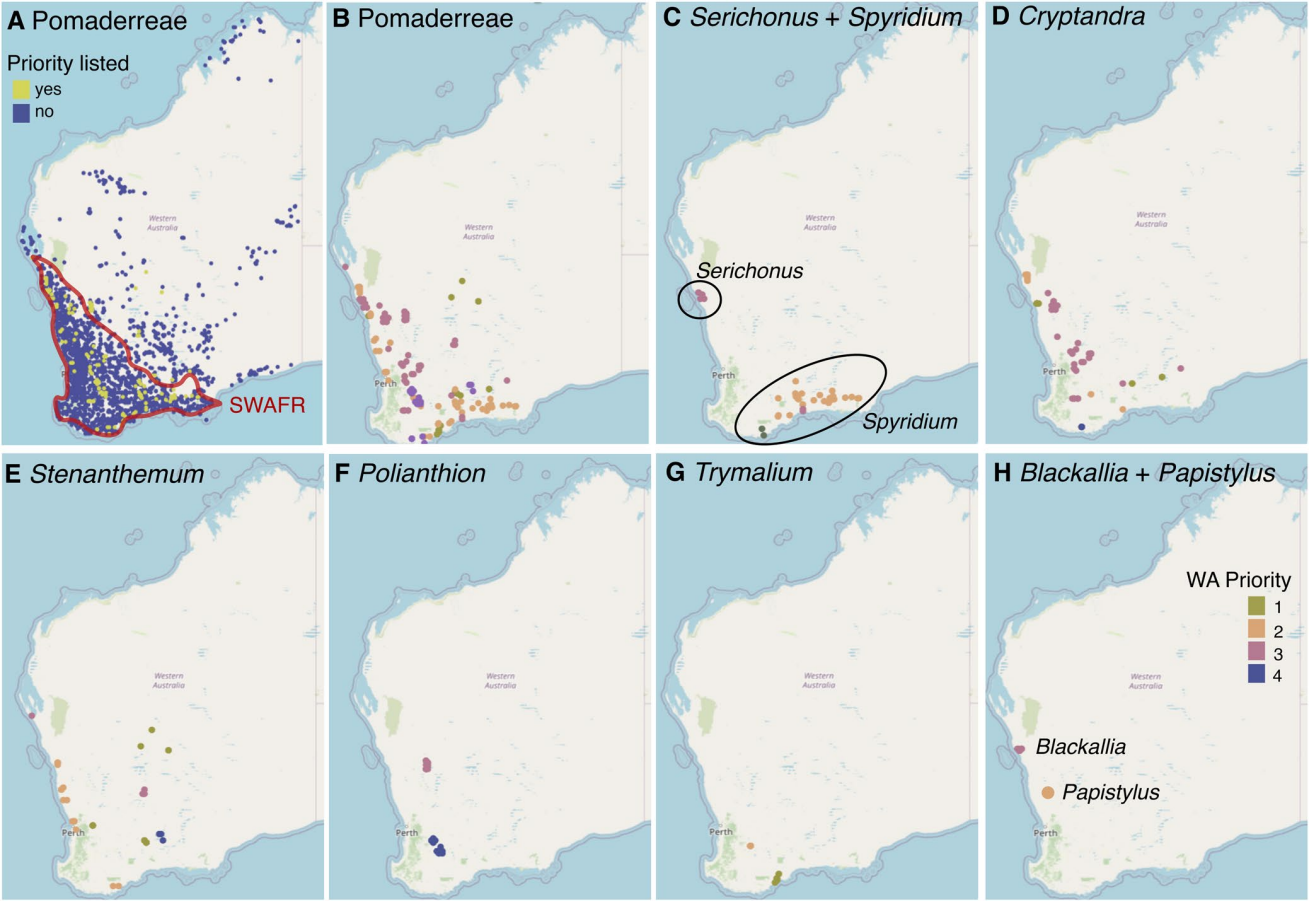
Maps showing the distribution points based on cleaned herbarium records of (A) Western Australian priority listed (yellow) and non‐priority listed (blue) Pomaderreae taxa and priority listed taxa colored according to the four priority levels: (B) all of Pomaderreae, (C) *Serichonus* + *Spyridium*, (D) Cryptandra, (E) Stenanthemum, (F) *Polianthion*, (G) *Trymalium*, (H) *Blackallia* + *Papistylus*. The southwest Western Australian Floristic Region (SWAFR) biodiversity hotspot is outlined in red.

We apologize for this error.

