# Supplementary material for: Environmental and Evolutionary Correlates of Extinction Risk in a Global Biodiversity Hotspot
Source: Ecol Evol. 2026 Aug 27;16(9):e74233. doi: 10.1002/ece3.74233 (PMC13519961; doi:10.1002/ece3.74233)
Supplement: Supplementary file 1 [file ECE3-16-e74233-s001.docx]

**Supplementary Materials:**

**Environmental and evolutionary correlates of extinction risk in a global biodiversity hotspot**

**Table S1.** Summary of the D-statistic tests for phylogenetic signal of extinction risk. The non-significant p-value for a random expectation indicates that the observed pattern is not strongly different from a random distribution across the phylogeny. The significant p-value (p < 0.05) under Brownian structure indicates the trait does not follow a Brownian phylogenetic pattern. NT = non-threatened.

| **Statistic** | **BINARY P4 as threatened** | **BINARY P4 as non-threatened (NT)** |
| --- | --- | --- |
| Binary variable analyzed | ext.risk | ext.risk |
| State counts | 0 = 58, 1 = 29 | 0 = 61, 1 = 26 |
| Number of permutations | 10,000 | 10,000 |
| Estimated D statistic | 0.8591 | 0.8074 |
| p-value (no phylogenetic structure; random expectation) | 0.2314 | 0.1688 |
| p-value (Brownian motion expectation) | 0.0037 | 0.007 |

| **Statistic** | **P1 vs. NT** | **P2 vs. NT** | **P3 vs. NT** | **P4 vs. NT** |
| --- | --- | --- | --- | --- |
| Binary variable analyzed | ext.risk | ext.risk | ext.risk | ext.risk |
| State counts | 0 = 80, 1 = 7 | 0 = 79, 1 = 8 | 0 = 76, 1 = 11 | 0 = 84, 1 = 4 |
| Number of permutations | 10,000 | 10,000 | 10,000 | 10,000 |
| Estimated D statistic | 0.5529 | 1.2883 | 0.8571 | 1 |
| p-value (no phylogenetic structure; random expectation) | 0.0951 | 0.8153 | 0.2846 | 0.6562 |
| p-value (Brownian motion expectation) | 0.2216 | 0.005 | 0.0387 | 0.1056 |

**Table S2.** Summary statistics of phylogenetic signal for flowering time (sum of months) using Blomberg’s K and Pagel’s lambda.

|  | **flowering time (sum of months)** | | |
| --- | --- | --- | --- |
| **Phylogenetic signal test** | **phylo signal** | **p-value** | **logL (lambda)** |
| Blomberg's K (nsim = 1000) | 0.4504 | 0.074 | NA |
| Pagel's lambda | 0.2057 | 0.101 | -155.94 |
